# MET Receptor Tyrosine Kinase Regulates Lifespan Ultrasonic Vocalization and Vagal Motor Neuron Development

**DOI:** 10.3389/fnins.2021.768577

**Published:** 2021-11-04

**Authors:** Anna K. Kamitakahara, Ramin Ali Marandi Ghoddousi, Alexandra L. Lanjewar, Valerie M. Magalong, Hsiao-Huei Wu, Pat Levitt

**Affiliations:** ^1^Program in Developmental Neuroscience and Neurogenetics, Children’s Hospital Los Angeles, The Saban Research Institute, Los Angeles, CA, United States; ^2^Department of Pediatrics, Keck School of Medicine of University of Southern California, Los Angeles, CA, United States; ^3^Neuroscience Graduate Program, University of Southern California, Los Angeles, CA, United States

**Keywords:** nucleus ambiguus, ultrasonic vocalization, MET receptor tyrosine kinase, vagus, development

## Abstract

The intrinsic muscles of the larynx are innervated by the vagal motor nucleus ambiguus (nAmb), which provides direct motor control over vocal production in humans and rodents. Here, we demonstrate in mice using the *Phox2b*^*Cre*^ line, that conditional embryonic deletion of the gene encoding the MET receptor tyrosine kinase (MET) in the developing brainstem (cKO) results in highly penetrant, severe deficits in ultrasonic vocalization in early postnatal life. Major deficits and abnormal vocalization patterns persist into adulthood in more than 70% of mice, with the remaining recovering the ability to vocalize, reflecting heterogeneity in circuit restitution. We show that underlying the functional deficits, conditional deletion of *Met* results in a loss of approximately one-third of MET^+^ nAmb motor neurons, which begins as early as embryonic day 14.5. The loss of motor neurons is specific to the nAmb, as other brainstem motor and sensory nuclei are unaffected. In the recurrent laryngeal nerve, through which nAmb motor neurons project to innervate the larynx, there is a one-third loss of axons in cKO mice. Together, the data reveal a novel, heterogenous MET-dependence, for which MET differentially affects survival of a subset of nAmb motor neurons necessary for lifespan ultrasonic vocal capacity.

## Introduction

The ability to vocalize is an essential form of communication in nearly all vertebrates (Barkan and Zornik, 2020). Analysis of laboratory rodent ultrasonic vocalizations (USVs) has served as a powerful model system for understanding the neural circuits underlying vocal communication, social and affiliative behaviors, and neurodevelopmental disorders (Hofer, 1996; Holy and Guo, 2005; Scattoni et al., 2009; Fischer and Hammerschmidt, 2011; Wöhr and Schwarting, 2013; Portfors and Perkel, 2014; Segbroeck et al., 2017). Much of the circuitry responsible for innate vocalization (e.g., rodent USVs, cries, and other non-verbal emotional utterances) is evolutionarily conserved in mammals, and develops prenatally, allowing infants and rodent pups to vocalize readily after birth (Hofer, 1996; Roubertoux et al., 1996; Arriaga et al., 2012; Hernandez-Miranda et al., 2017; Barkan and Zornik, 2020). Identifying the precise molecular instructive signals that are required for the development of neural-mediated vocal function will provide a foundational understanding of the circuitry underlying vocal production and has implications for understanding how communication deficits arise.

A few studies have begun to dissect early genetic and molecular factors that are necessary for the development of vocalization circuits. Constitutive loss or functional mutations of *Foxp2* result in the complete absence of USVs in early postnatal mice and are associated with multilevel alterations in the cortex, striatum, and cerebellum (Shu et al., 2005; French et al., 2007, 2012; Spiteri et al., 2007; Fujita et al., 2008; Groszer et al., 2008; Chabout et al., 2016; Urbanus et al., 2020). Additionally, deletion of *Olig3* or *Tlx3* result in the loss of large portions of the nucleus tractus solitarius (NTS) and dramatic reductions in early postnatal USVs (Hernandez-Miranda et al., 2017). The causality is unclear, however, because these phenotypes occur prior to the onset of respiratory impairments in these mice, which leads to lethality within the first 12 h of birth (Hernandez-Miranda et al., 2017). Deletion of *Tshz3* reveals that its expression is critical for both development of respiratory neurons and survival of a large portion of the nucleus Ambiguus (nAmb), where phonatory laryngeal motor neurons reside (Caubit et al., 2010). We reported previously in prenatal mice that highly restricted subsets of developing medullary neurons in the NTS and larynx-projecting nAmb motor neurons express MET (Wu and Levitt, 2013; Kamitakahara et al., 2017), positioning this receptor tyrosine kinase as a candidate for influencing the development of vocalization circuits.

MET is a pleiotropic receptor that is important for synapse maturation and critical period regulation in the cerebral cortex (Xie et al., 2016; Ma and Qiu, 2019; Chen et al., 2020) and for the differentiation of several different motor neuron pools in the brainstem and spinal cord (Ebens et al., 1996; Wong et al., 1997; Caton et al., 2000; Tallafuss and Eisen, 2008). Upon binding to its only known ligand, hepatocyte growth factor (HGF), MET confers neuronal survival in developing spinal motor neurons that innervate the pectoralis minor muscle (Lamballe et al., 2011). By contrast, in adjacent limb-innervating motor neurons, MET is dispensable for neuronal survival but instead stimulates axonal elaboration (Lamballe et al., 2011). A recent study in zebrafish shows that similar to its role in spinal motor neurons, MET signaling is involved in axon growth and guidance of vagal motor neurons that innervate the pharyngeal arches (Isabella et al., 2020). Mammalian species exhibit considerable differences from fish in vagal anatomy and pharyngeal arch-derived structures, as mammals form a larynx instead of gill arches. However, homologous to patterns observed in zebrafish, HGF is expressed embryonically in the developing airways of mice (Kamitakahara et al., 2017). This suggests that in mammals, MET may serve as a signal for instructing the development of medullary vagal laryngeal neurons.

Here, we used a conditional knockout strategy to delete MET from vagal motor neurons in mice expressing Cre recombinase under the control of the Phox2b promoter (cKO mice). Using this transgenic mouse model, in combination with several transgenic reporter lines, the functional and neuroanatomical effects of conditional deletion of MET on USV production and nAmb development were examined. These studies reveal a novel role for MET signaling in vagal motor neuron development and functional vocalization across the lifespan.

## Materials and Methods

### Animals

Animal care and experimental procedures were performed in accordance with the Institutional Animal Care and Use Committee of The Saban Research Institute, Children’s Hospital Los Angeles. Mice were housed in the vivarium on a 13:11 h light:dark cycle (lights on at 06:00 h, lights off at 19:00 h) at 22°C with *ad libitum* access to a standard chow diet (PicoLab Rodent Diet 20, #5053, St. Louis, MO).

A number of genotypically unique strains were generated through the mating of available transgenic mice in order to perform the current studies. The *Phox2b*^*cre*^ and Cre-dependent TdTomato reporter lines (*TdTom*) were obtained from The Jackson Laboratory [B6(Cg)-Tg(Phox2b-cre)3Jke/J, stock 016223, RRID:IMSR_JAX:016223 and B6.Cg-Gt(ROSA)26Sortm14(CAG-tdTomato)Hze/J, stock 007914; Ai14, RRID:IMSR_JAX:007914, respectively]. Initial validation studies were performed to assess recombination efficiency in the vagal motor nuclei using *Phox2b^*cre*^; TdTom* mice. The TdTom reporter was used as a marker of recombination and choline acetyltransferase (ChAT) was used as a marker to identify all vagal motor neurons in the dorsal motor nucleus of the vagus (DMV) and nAmb. Nearly 100% of ChAT+ neurons were also TdTom+, demonstrating high recombination efficiency in the vagal motor nuclei using the *Phox2b*^*cre*^ line (data not shown). We employed a strategy of utilizing the *Phox2b*^*cre*^ driver line with the *Met*^*fx*^ line to leverage the highly selective expression overlap in vagal motor neurons, very limited overlap in NTS neurons, and no overlap in nodose (sensory) ganglion neurons.

The *Met*^*fx*^ mouse line, in which exon 16 of the *Met* allele is flanked by loxP sites, was shared by the laboratory of Dr. Snorri S. Thorgeirsson (National Cancer Institute, NIH, Bethesda, MD) and is available at the Jackson Laboratory (Stock 016974). *Met*^*EGFP*^ mice were generated as previously described (Kamitakahara et al., 2017; Kast et al., 2017) using GENSAT project clone BX139 (Rockefeller University, RRID:SCR_002721) (Geschwind, 2004). In this mouse model, the EGFP transcript is downstream of the MET promoter, and does not result in additional MET receptor expression because of the insertion. Expression concordance between *Egfp* and *Met* transcript or protein in neurons residing in the neocortex, raphe and vagal motor complex is nearly 100% in *Met*^*EGFP*^ mice. Both the *Met*^*fx*^ and *Met*^*EGFP*^ transgenic lines have been backcrossed for more than 10 generations and maintained isogenically on a C57BL/6J background in our laboratory for all experiments described.

To simplify the strain names of mice used, Cre negative (Cre-) will be used to describe any mouse lacking the *Phox2b*^*cre*^ allele, conditional wild type (cWT) will be used to describe any mouse carrying the *Phox2b*^*cre*^ allele but lacking the *Met*^*fx*^ allele, and conditional knockout (cKO) will be used to describe any mouse carrying the *Phox2b*^*cre*^ allele and two copies of the *Met*^*fx*^ allele. A complete listing of genotypes that fall under the Cre-, cWT, and cKO designations is included in Supplementary Table 1.

### Ultrasonic Vocalization

Isolation-evoked USVs were recorded on P7 using a CM16/CMPA ultrasound microphone positioned 5 cm above the recording chamber floor, and an UltraSoundGate 116H recorder (Avisoft Bioacoustics). Mice were maintained in the home nest environment in a cage warmed by a heating pad until the moment of recording. For each 5-min recording, a single pup was removed from the nest and recorded in isolation without heat support in the recording chamber. Following each recording, an additional 15-s recording was made to determine whether USVs could be evoked by acute tail pinch. Computed values for USV number and duration were determined using Avisoft-SAS Lab Pro software (Avisoft Bioacoustics, RRID:SCR_014438). Genotype was unknown to the operator.

Adult male USVs were recorded during a direct social interaction task using a CM16/CMPA ultrasound microphone positioned 16 cm above the chamber floor, and an UltraSoundGate 116H recorder (Avisoft Bioacoustics). Prior to the day of testing, males from each genotype group were exposed to a 3–4-month old C57BL/6J female partner for 3 days in their home cage to gain social experience. Females were then removed from the cage, and males were housed in isolation for 2 days to increase motivation to call during the direct social interaction task. On the day of testing, males were habituated to the recording chamber for 10 min, then recorded for 6 min with a novel 3–4-month old C57BL/6J female. Computed values for USV number and duration were determined using Avisoft-SAS Lab Pro software (Avisoft Bioacoustics, RRID:SCR_014438). Genotype was unknown to the operator.

### Respiratory Analysis

USV recordings from P7 mouse pups were analyzed based on parameters determined by Sirotin et al. (2014) and used by Urbanus et al. (2020) to examine respiratory patterns of mouse pups during vocalization. To specifically examine respiratory pattern during vocalization, analysis was confined to vocalizations within a bout, defined as a string of 2 or more calls or clicks (e.g., events) made within 300 ms of each other. Sirotin et al. (2014) demonstrated that USVs that occur within 60 ms of each other are produced within the same breath. To estimate the number of multi-event breaths, the number of events within 60 ms of one another was quantified. Within a bout, the average time between events, or inter-syllable-interval was quantified as a measure of the inspiratory pattern during respiration.

### Mice Ultrasonic Profile ExTractor Analysis

To assess possible alterations in specific patterns of vocalization, Mice Ultrasonic Profile ExTractor (MUPET) v2.0 (Segbroeck et al., 2017), an open-access MATLAB (MATLAB_R2021a) NeuroResource, was used to analyze experience-evoked USVs recorded on P7 and P60. MUPET uses a complex clustering algorithm to categorize individual USV syllables into a “repertoire unit,” based on syllable shape (Segbroeck et al., 2017). The collection of all the repertoire units made by each genotype or group is referred to as a “repertoire.” Audio files were processed in MUPET, and a dataset was created for each genotype or genotype subset. At each age and for each genotype, repertoires of 20–140 repertoire units were built to determine the appropriate repertoire size best suited for the analysis. For P7, a repertoire size of 60 units was determined to be optimal based on an overall repertoire modeling score, average log-likelihood, Bayesian Information Criterion, and the number of repertoire units containing only one syllable. The same criteria were used at P60 to determine 100 units as the optimal repertoire size at this age. The “Best Match Sorting” feature was used to generate matrices of Pearson correlations, comparing the similarity of each repertoire unit from the Cre- dataset to repertoire units in all other genotype or genotype subset datasets. The “Unit Activity Sorting” feature was used to generate a Pearson correlation coefficient for overall repertoire similarity. To estimate the Pearson correlation coefficient for the Cre- repertoire, six jackknife resampled datasets were analyzed using MUPET (Quenouille, 1956; Efron, 1982). These jackknife resampled datasets were generated by systematically leaving out one recording in each dataset from the three highest and three lowest vocalizers. Together, the Best Match Sorting and Unit Activity Sorting features measure the similarity of individual repertoire units, and the overall similarity of repertoires between groups, respectively.

### Immunohistochemistry

Tissue for immunofluorescence staining was collected on embryonic days (E) 9.5 and 14.5, postnatal day (P) 7, and in P60-P80 (denoted elsewhere as P60 or adult) mice. For embryonic tissue collection, timed pregnant breeding pairs were set in the evening, and observance of a vaginal plug the following morning was designated as E0.5. Sex was not determined for embryonic samples. Embryonic tissue was collected and immersed overnight in fixative [4% paraformaldehyde in 0.1 M phosphate-buffered saline (PBS, pH 7.4)]. Postnatal mice were deeply anesthetized by intraperitoneal injection of ketamine:xylazine (100 mg/kg:10 mg/kg, Henry Schein, Melville, NY) and perfused transcardially with 0.9% saline, followed by fixative. Collected tissues were postfixed, cryoprotected in 20% sucrose in PBS, embedded in Tissue-Tek^®^ Optimal Cutting Temperature Compound, and frozen over liquid nitrogen vapors or powdered dry ice. For brain tissue, 20 μm-thick cryostat sections were collected in five coronal or two sagittal series representing the entire rostral-caudal or medial-lateral extent of the nAmb. For laryngeal tissue, 30 μm-thick cryostat sections were collected in five coronal series. For recurrent laryngeal nerve (RLN) axon cross sections, 10 or more 20 μm coronal sections were collected between the fifth tracheal ring and the larynx. Slides were stored at −20°C until processed.

For immunofluorescence labeling, slides were blocked and then incubated in a solution containing one or more of the following primary antibodies: chicken anti-Green Fluorescent Protein (GFP) (1:500, Abcam Cat# ab13970, RRID:AB_300798), goat anti-MET receptor tyrosine kinase (MET) (1:500, R and D Systems Cat# AF527, RRID:AB_355414), mouse monoclonal anti-Neurofilament H 1G9 (NF) (1:500, Pennypacker et al., 1991), or rabbit anti-Red Fluorescent Protein (RFP) (1:750, Rockland Immunochemicals Cat# 600-401-379). Sections were washed in PBS, then incubated in solution with one or more of the following secondary or tertiary antibodies: Alexa Fluor^®^ 488 AffiniPure F(ab’)_2_ Fragment Donkey Anti−Chicken IgG (Jackson ImmunoResearch Cat# 703–546−155, RRID:AB_2340376), Biotin−SP−AffiniPure F(ab’)_2_Fragment Donkey Anti−Goat IgG (Jackson ImmunoResearch Cat# 705–066−147, RRID:AB_2340398), Alexa Fluor^®^ 488 Streptavidin (Jackson ImmunoResearch Cat# 016-540-084, RRID: AB_2337249), Alexa Fluor^®^ 647 AffiniPure Donkey Anti-Mouse IgM, μ chain specific (Jackson ImmunoResearch Cat# 715-605-020, RRID: AB2340860), or Alexa Fluor^®^ 594 AffiniPure F(ab’)_2_ Fragment Donkey Anti−Rabbit IgG (Jackson ImmunoResearch Cat# 711-586-152, RRID:AB_2340622). To label acetylcholine receptor clusters in muscular tissue, α-Bungarotoxin (αBT) Alexa Fluor^®^ 488 conjugate (Thermo Fisher Scientific Cat# B13422) was added to the secondary antibody solution. Sections were counterstained with DAPI (Thermo Fisher Scientific Cat# D1306) and embedded with ProLong Gold Antifade Mountant (Thermo Fisher Scientific Cat# P36930) prior to applying a coverslip.

### Image Acquisition

A Zeiss LSM 710 laser scanning confocal microscope equipped with 10×, 20×, 40× water-corrected, and 63× oil-corrected objectives was used to acquire immunofluorescence images. Confocal image stacks were collected through the *z*-axis at a frequency optimally determined by the Zeiss Zen software based on the optics of the microscope and the wavelength of the fluorophores used for analysis. Slides were coded so that the operators were blind to experimental group.

For cell counts of EGFP+ and tdTomato+ neurons in the nAmb, DMV, and NTS, every fifth consecutive coronal section and second sagittal section was imaged through the entire rostral-caudal or medial-lateral length of the nucleus using a 20× objective. For imaging of the RLN and laryngeal muscle innervation, anatomical regions of interest for each muscle or nerve were identified, and confocal image stacks were captured using a 20×, 40× water-corrected, or 63× oil-corrected objective.

### Image Analysis

For cell counts of tdTomato+ and EGFP+ neurons in the nAmb, DMV, or NTS, all image stacks for each animal were manually analyzed using the “Cell Counter” plugin within the Fiji/ImageJ software. The boundaries of the nAmb and cell inclusion/exclusion criteria were agreed upon by two different experimenters prior to cell counts. For tdTomato+ counts, all neurons within each medullary nucleus that had DAPI colocalized with tdTomato were counted. For EGFP+ counts, all neurons within the medullary nucleus that had DAPI colocalized with both tdTomato and EGFP were counted. Diameters of at least 5% of nuclei were measured in Fiji/ImageJ and averaged per animal. The total number of cells in the nAmb, DMV, or NTS of each animal was estimated in accordance with Abercrombie’s formula (Abercrombie, 1946). Count validation was established by two operators independently counting samples.

For the generation of sagittal topology maps, each cell position was labeled with a region of interest (ROI) in Fiji/ImageJ and the ROIs were uploaded into R Studio using the *RImageJROI* package. The X and Y coordinates of each cell ROI were then aggregated and plotted onto a reference coordinate denominated by the most dorsal-caudal point of the facial nucleus. Mapped cellular coordinates from each section and animal were aggregated by group and plotted to generate reconstructed topological maps of the nucleus.

For analyses of laryngeal muscle innervation, postsynaptic αBT labeled acetylcholine receptor clusters were counted using the “Cell Counter” plugin in Fiji/ImageJ. The number of αBT labeled clusters closely apposed to tdTomato labeled fibers were then counted to generate a measurement of percent innervation.

For analysis of RLN cross sections, each image was collapsed into a maximum intensity z-stack in Fiji/ImageJ, a rolling-ball radius (rbr) background removal step was performed (“Subtract background” with rbr of 15; “Disable Smoothing”), the axon bundle was manually circled, and the “Clear Outside” option was used to remove non-specific signal inside and outside of the nerve bundle. Single axon fibers were quantified in an automated manner using the “StarDist” plugin (DSB 2018 model; Probability Threshold of 0.4; Overlap Threshold of 0.0; Schmidt et al., 2018).

### Quantification and Statistical Analysis

Data were analyzed statistically and graphed using GraphPad Prism software (RRID:SCR_002798) and expressed as mean values ± standard error of the mean. The number of animals required for quantitative analysis was calculated based on power analysis, with the aim of detecting differences between groups that were 1–2 standard deviations from the mean with at least 80% power and *p* < 0.05 for significance. Each mouse is considered a sample, with sample sizes of each genotype included in the figure legends for each analysis. For each genotype, a D’Agostino-Pearson normality test was used to determine whether parametric or non-parametric statistical analyses should be performed. For data following a normal distribution, an ordinary one-way ANOVA or a parametric two-tailed unpaired *t*-test was used to compare means. For data that failed to pass the D’Agostino-Pearson normality test, a non-parametric Kruskal-Wallis test (correcting for multiple comparisons using Dunn’s test) or a non-parametric two-tailed Mann-Whitney test was used to compare mean rank difference. Due to small sample sizes, normality tests were not performed for the sagittal counts, and a *t*-test was used to compare means. To compare correlation coefficients, Fisher’s r-to-z transformation was applied followed by *z*-tests with Bonferroni correction. A *p* < 0.05 was used for significance. The individual statistical tests used and sample sizes are further indicated in each figure legend.

## Results

### Ultrasonic Vocalization Is Impaired in Early Postnatal Life Following Loss of MET

**FIGURE 1:**
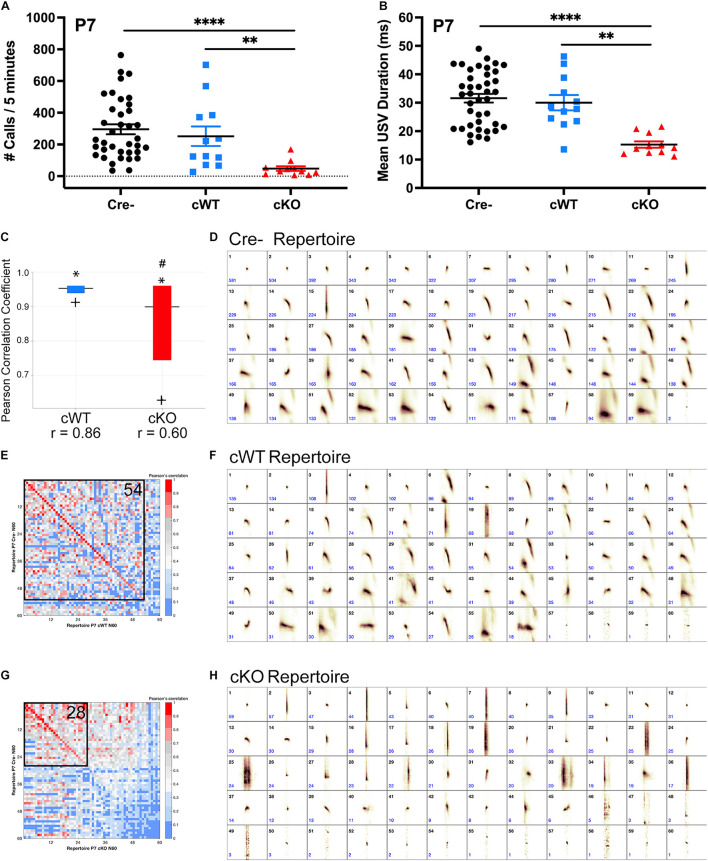
Deletion of MET results in severely impaired ultrasonic vocal production and syllable repertoire early postnatally. **(A)** Quantification of the number of isolation-evoked USVs over the 5-min recording period on P7. *n* = 37 Cre-, *n* = 12 cWT, *n* = 11 cKO. “^∗∗^” indicates *p* < 0.01, “^****^” indicates *p* < 0.0001 as analyzed by non-parametric Kruskal-Wallis test and Dunn’s correction for multiple comparisons. **(B)** Quantification of the duration of isolation-evoked USVs over the 5-min recording period on P7. *n* = 37 Cre-, *n* = 12 cWT, *n* = 11 cKO. “^∗∗^” indicates *p* < 0.01, “^****^” indicates *p* < 0.0001 as analyzed by non-parametric Kruskal-Wallis test and Dunn’s correction for multiple comparisons. **(C)** MUPET boxplot comparing the Cre- repertoire to the cWT repertoire (blue) or cKO repertoire (red). Similarity of the top 5% most frequently used RUs in the Cre negative repertoire indicated by ^∗^. Similarity of the top 25% most frequently used RUs in the Cre negative repertoire indicated by top of the box. Similarity of the top 50% most frequently used RUs in the Cre- repertoire indicated by the horizontal line. Similarity of the top 75% most frequently used RUs in the Cre- repertoire indicated by the bottom of the box. Similarity of the top 95% most frequently used RUs in the Cre- repertoire indicated by +. *r*-values below boxes indicate the overall Pearson correlation coefficient for the entire repertoire. # indicates overall Pearson’s *r*-values that are significantly different from the Cre- repertoire analyzed using a Fisher r-to-z transformation to make pairwise *p*-value calculations followed by Bonferroni correction for multiple comparisons. **(D)** Each repertoire unit (RU) in the Cre- repertoire, displayed in the order of frequency of use. **(E,G)** Pearson correlation matrices comparing each of the Cre- RUs (*y*-axis) to RUs in the cWT repertoire (*x*-axis, **E**) or cKO repertoire (*x*-axis, **G**), ordered from most to least similar in shape. Warmer colors indicate higher Pearson correlation, cooler colors indicate lower Pearson correlation. Boxed area shows the number of RUs with Pearson correlations above 0.7, with the corresponding number of RUs indicated in the upper right corner. **(F)** Each repertoire unit (RU) in the cWT repertoire, displayed in the order of frequency of use. **(H)** Each repertoire unit (RU) in the cKO repertoire, displayed in the order of frequency of use. Total syllable number in each MUPET repertoire: Cre- = 12,070; cWT = 3,416; cKO = 1,196.

The activity of vagal laryngeal motor neurons in the nAmb is required for rodent ultrasonic vocalization (USV) (Wetzel et al., 1980; Yajima et al., 1982; Nunez et al., 1985). To examine whether expression of MET by vagal neurons is necessary for vocal function, we used a Cre-Lox strategy to conditionally delete *Met* using a histologically validated *Phox2b^*cre*^; Met^*fx/fx*^* (cKO) transgenic mouse model (Supplementary Figures 1-1, 1-2; Huh et al., 2004), in which there is extensive overlap in expression of both genes in specific vagal motor neuron subtypes (Scott et al., 2011; Kamitakahara et al., 2017). To test whether vagal motor output is functionally altered following loss of MET, cKO mice were used to examine USVs evoked by a standard method of brief isolation of mouse pups from the nest recorded on postnatal day (P) 7. Following removal from the nest, control (Cre-) pups made robust USV calls over the course of a 5-min recording period. Both the number and duration of calls were similar between Cre- and conditional wild-type (cWT) control pups, demonstrating that Cre expression alone in this transgenic model has no effect on vocalization (Figures 1A,B). By contrast, cKO mutant pups exhibited severely reduced numbers of calls and decreased call duration (Figures 1A,B), indicating that the loss of MET in vagal neurons impairs USV production. Remarkably, at this age, the USV disrupted phenotype was fully penetrant for all cKO mice tested.

To determine whether the loss of isolation-evoked USVs is due to a motor deficit to call, or perhaps a reduced motivation to call, short 15-s recordings were made following a brief tail pinch stimulus to directly elicit vocalization. The mechanical tail pinch stimulus provokes an involuntary painful vocalization in mouse pups. Therefore, a lack of USVs evoked by tail pinch would further demonstrate motor deficits in the ability to call. While both Cre- and cWT pups evoked many complex USVs following the tail pinch, cKO pups made very few USVs, if any, and those emitted were very short in duration (Supplementary Figures 1-3A,D). cKO mice also did not make any calls in the audible range, but did produce significantly more clicks (a straight vertical shape on the spectrogram) than Cre- pups (Supplementary Figures 1-3B–D), suggesting that emitted clicks may be generated by cKO pups in place of audible calls and complex USVs. Together, these results demonstrate that pup vocalization is severely impacted by loss of MET in vagal neurons.

To examine whether the loss of MET results in major changes in respiratory patterns during vocalization, a secondary analysis of USV recordings was performed. Mice typically take a single breath between each call, but occasionally will make more than one call in the same breath (Sirotin et al., 2014). The ability to produce these multi-event breaths are indicative of respiratory control. To examine irregularities in breathing pattern during vocalization, the number of breaths that have more than one call or click (e.g., event) were analyzed in cWT and cKO pups. Although cKO pups vocalize much less, for those bouts that did occur, there were no differences in the percentage of multi-event breaths. This analysis indicates that cKO pups possess sufficient respiratory control necessary to make multiple calls within a single breath (Supplementary Figure 1-4A). Furthermore, within a call bout, there was no difference in the inter-event-interval (Supplementary Figure 1-4B), suggesting that the timing of the call-breath-call respiratory pattern is similar between genotypes.

There was a small, but significant decrease (∼20%) in the body weight of mutant cKO pups on P7 (Supplementary Figure 1-5A). To determine if such changes impacted vocalization capacity, the number of calls were examined as a function of pup body weight. The analysis revealed that both Cre- and cWT pups of comparable body weights to the knockout mice readily emitted isolation-evoked USVs, suggesting that the inability of cKO pups to make calls is not a result of being smaller (Supplementary Figure 1-5B). Furthermore, there were no differences in body weight in adult males and females between genotypes (Supplementary Figures 1-5C,D).

Rodent USV repertoires are composed of a diverse compliment of syllable shapes used to generate strain, sex, and context-specific vocal communication across development and in adulthood (Holy and Guo, 2005; Grimsley et al., 2011; Arriaga et al., 2012; Segbroeck et al., 2017). Given that loss of MET results in a major reduction in the number and duration of calls made by cKO mice in early postnatal life, we assessed whether the shape of the few syllables emitted also might be altered, reflecting motor impairments in the ability to produce complex vocalizations. To analyze differences in USV repertoires between genotypes, Mouse Ultrasonic Profile ExTraction (MUPET) software was used to perform unsupervised signal processing of isolation-evoked USVs recorded on P7 (Segbroeck et al., 2017). Within each genotype, all recorded syllables were clustered into repertoires composed of 60 repertoire units (RUs). Each RU represents the syllable centroid, or average of syllable shapes in that RU cluster. The Cre- repertoire was composed of several distinct RUs (short simple, chevrons, frequency jumps, complex harmonics, etc.) (Figure 1D). The shape of each of these RUs in the Cre- repertoire was then statistically compared to RUs clustered in cWT and cKO repertoires (Figures 1F,H). For this analysis, MUPET generates a matrix of individual Pearson correlations for pairwise comparisons of RUs in each repertoire. The matrix is color coded such that warmer colors correspond to highly similar RUs. Fifty-four out of 60 RUs had Pearson correlations greater than 0.7 when comparing the cWT and Cre- repertoires (colored red to pink across the matrix diagonal in Figure 1E), demonstrating that the shape of the syllables in these repertoires are highly similar to one another. In contrast, the cKO repertoire was primarily composed of very short syllables, and a straight vertical shape consistent with emitted clicking sounds. Only 28 out of 60 RUs in the cKO repertoire had Pearson correlations greater than 0.7 when compared to the Cre- repertoire (Figure 1G) and were primarily made up of very short simple shapes, demonstrating the production of both a very limited and distinct syllable repertoire in the cKO mice. In addition to comparing individual RUs, the entire Cre- repertoire was compared to the entire cWT and cKO repertoires. Pearson correlation coefficients were calculated for 100% of the syllables produced in each repertoire. While the cWT repertoire was highly similar to the Cre- repertoire, with an overall Pearson’s r of 0.86, the cKO repertoire was significantly different from the Cre- repertoire, with an overall Pearson’s r of 0.60 (Figure 1C). These data indicate that expression of MET in vagal neurons is critical for generating the diversity and complexity of syllables in the vocal repertoire produced by P7 pups.

### Ultrasonic Vocalizations Are Impaired in the Majority of Male Conditional Knockout Mice in Adulthood

**FIGURE 2:**
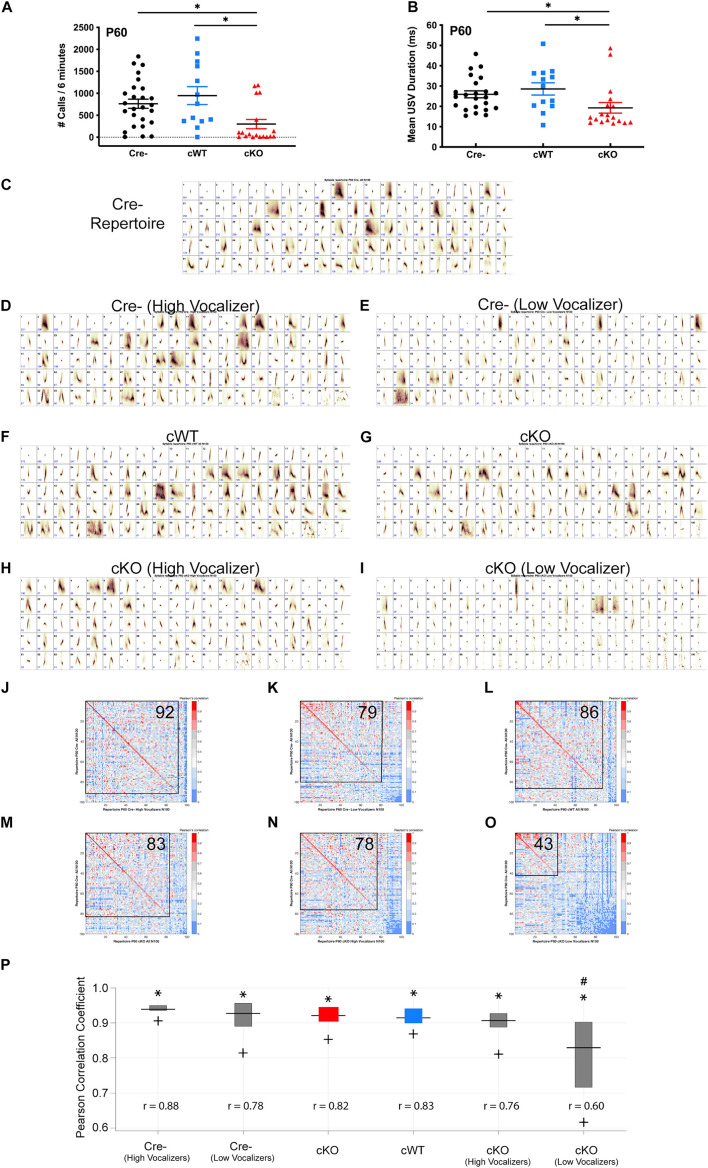
Sustained vocalization deficits in *Met* cKO adult mice. **(A)** Quantification of the number of USVs produced by P60 males paired with females over a 6-min recording period during a direct social interaction task. *n* = 26 Cre-, 13 cWT, 18 cKO. “^∗^” indicates *p* < 0.05 as analyzed by non-parametric Kruskal-Wallis test and Dunn’s test to correct for multiple comparisons. **(B)** Quantification of the duration of USVs made by P60 males over the 6-min recording period. *n* = 26 Cre-, 13 cWT, 18 cKO. “^∗^” indicates *p* < 0.05 as analyzed by non-parametric Kruskal-Wallis test and Dunn’s test to correct for multiple comparisons. **(C)** Each repertoire unit (RU) in the Cre- repertoire, displayed in the order of frequency of use. **(D)** Each repertoire unit (RU) in the Cre- high vocalizer repertoire, displayed in the order of frequency of use. Repertoire generated from 13 Cre- recordings with the highest number of calls. **(E)** Each repertoire unit (RU) in the Cre- low vocalizer repertoire, displayed in the order of frequency of use. Repertoire generated from 13 Cre Negative recordings with the lowest number of calls. **(F)** Each repertoire unit (RU) in the cWT repertoire, displayed in the order of frequency of use. **(G)** Each repertoire unit (RU) in the cKO repertoire, displayed in the order of frequency of use. **(H)** Each repertoire unit (RU) in the cKO high vocalizer repertoire, displayed in the order of frequency of use. Repertoire generated from 5 cKO recordings with the highest number of calls. **(I)** Each repertoire unit (RU) in the cKO low vocalizer repertoire, displayed in the order of frequency of use. Repertoire generated from 13 cKO recordings with the lowest number of calls. **(J–O)** Pearson correlation matrices comparing each of the Cre- RUs (*y*-axis) to RUs in the Cre- high vocalizer repertoire (*x*-axis, **J**), Cre- low vocalizer repertoire (*x*-axis, **K**), cWT repertoire (*x*-axis, **L**), cKO repertoire (*x*-axis, **M**), cKO high vocalizer repertoire (*x*-axis, **N**), or cKO low vocalizer repertoire (*x*-axis, **O**), ordered from most to least similar in shape. Warmer colors indicate higher Pearson correlation, cooler colors indicate lower Pearson correlation. Boxed area shows the number of RUs with Pearson correlations above 0.7, with the corresponding number of RUs indicated in the upper right corner. **(P)** MUPET boxplot comparing the Cre- repertoire to each group. Similarity of the top 5% most frequently used RUs in the Cre- repertoire indicated by ^∗^. Similarity of the top 25% most frequently used RUs in the Cre- repertoire indicated by top of the box. Similarity of the top 50% most frequently used RUs in the Cre- repertoire indicated by the horizontal line. Similarity of the top 75% most frequently used RUs in the Cre- repertoire indicated by the bottom of the box. Similarity of the top 95% most frequently used RUs in the Cre- repertoire indicated by +. *r*-values below boxes indicate the overall Pearson correlation coefficient for the entire repertoire. # Indicates overall Pearson’s *r*-values that are significantly different from the Cre- repertoire analyzed using a Fisher r-to-z transformation to make pairwise *p*-value calculations followed by Bonferroni correction for multiple comparisons. Total syllable number in each MUPET repertoire: All Cre- = 21,002; Cre- (High Vocalizer) = 14,651; Cre- (Low Vocalizer) = 6,351; cWT = 11,494; All cKO = 6,370; cKO (High Vocalizer) = 4,803; cKO (Low Vocalizer) = 1,567.

To determine whether the inability to emit USVs is sustained into adulthood, separate cohorts of Cre-, cWT, and cKO male mice were examined at P60 using a standard direct social interaction task with a wild-type female C57BL/6J conspecific. Only males were analyzed in this paradigm, as females did not produce USVs in sufficient numbers in this behavioral paradigm to distinguish from baseline (data not shown) (Heckman et al., 2016). Both Cre- and cWT males averaged several hundred calls over the course of the 6-min recording period (Figure 2A). By contrast, cKO male mice produced significantly fewer USVs over that same time period (Figure 2A). Furthermore, average USV call duration was significantly reduced in mutant mice (Figure 2B). Intriguingly, the responses of cKO male mice appeared to follow two distinct patterns: more than 70% of mice tested exhibited very limited vocalizations during interaction with the females, using only short simple syllables (cKO low vocalizers); a smaller group of mice vocalized fairly robustly and were able to produce more complex syllables (cKO high vocalizers). These data, together with the nearly fully penetrant vocalization phenotype at P7, indicate that while the majority of adult cKO mice continued to have a very limited ability to vocalize, a small subset (5/18; 27.8%) appeared to regain USV-related laryngeal function.

To determine whether USV repertoire impairments observed in early postnatal life are sustained into adulthood, MUPET was used to examine syllable repertoires in recordings from P60 males paired with females in the direct social interaction task. Within each genotype, all recorded syllables were clustered into 100 RUs, based on modeling parameters. The Cre- repertoire was composed of a diverse array of simple and complex syllable shapes (Figure 2C). cWT and cKO repertoires were composed of syllables with high similarity (Pearson’s correlations above 0.7) to the Cre- repertoire, 86/100 and 83/100, respectively (Figures 2F,G,L,M). Given observations that the USV recordings from cKO mice at P60 were either low vocalizers or high vocalizers, repertoires of these sub-sampled groups were compared. USV recordings from cKO mice were grouped into either low (*n* = 13) or high vocalizing (*n* = 5) groups split by the mean number of USVs in that genotype. Leveraging the vocalization variation in control mice, Cre- mice were also grouped into either low or high vocalizing groups split by the mean number of USVs in that genotype. When each of these four subgroups was compared to the entire Cre- repertoire, it was found that both Cre- high and low vocalizers had high Pearson correlations for syllable shape when compared to the entire Cre- group (Figures 2D,E,J,K). These data indicate that the syllables produced by control, low vocalizing mice are very similar to those produced by control, high vocalizing mice. Similarly, 78/100 syllables from the five high vocalizing cKO mice exhibited high Pearson correlations for syllable shape (Figures 2H,N), indicating that both the frequency and quality of vocalizations in this small group of mice were normal, even in the absence of MET. In contrast, for the cKO low vocalizer group, only 43/100 syllables had Pearson correlations above 0.7 (Figures 2I,O), revealing both that the vocalizations were less frequent and that the repertoires of these mice were distinct from the Cre- controls. Similar to recordings at P7, the cKO low vocalizer recordings were composed primarily of very short syllables with a straight vertical shape that is often associated with either a clicking sound or noise artifact. The entire Cre- repertoire also was compared to all repertoires from other genotypes by calculating Pearson correlation coefficients for 100% of used syllables. Both Cre- high and low vocalizers had large Pearson correlations for syllable shape when compared to the entire Cre- group, 0.87 and 0.80, respectively (Figure 2P). Similarly, cWT, cKO, and cKO high vocalizers had high overall Pearson correlations coefficients when compared to the full Cre- repertoire (Figure 2P). However, cKO low vocalizers had a significantly smaller Pearson’s *r*-value (Figure 2P). This demonstrates that the apparently “normal” repertoire of the entire cKO group is being driven by the few high vocalizers, while major deficits in USVs are sustained into adulthood in most cKO mice.

### Reduced Recurrent Laryngeal Branch Axons Following Deletion of Met in Nucleus Ambiguus

**FIGURE 3:**
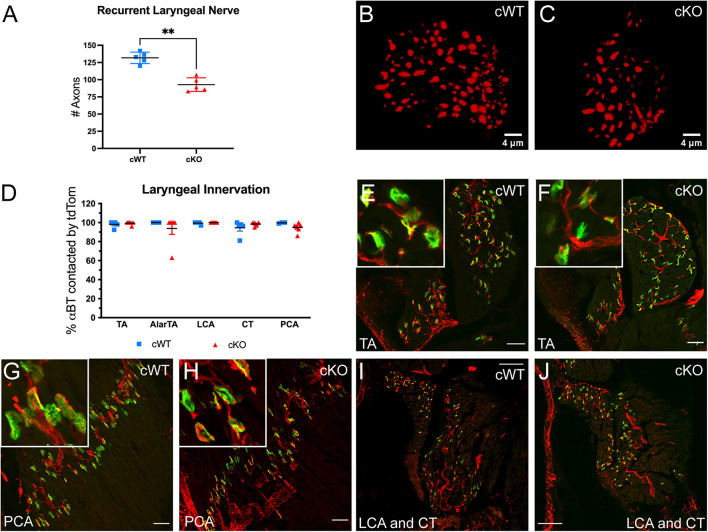
Axonal quantification and innervation status of αBT labeled AChR clusters in laryngeal muscles following MET deletion. **(A)** Quantification of the number of axons in the recurrent laryngeal nerve in cWT and cKO mice on P7. *n* = 5 cWT, 5 cKO. *t*-test, “^∗∗^” indicates *p* ≤ 0.05. **(B,C)** Representative images of recurrent laryngeal nerve cross sections labeled with neurofilament (red) in cWT and cKO mice on P7. Scalebars = 4 μm. **(D)** Quantification of laryngeal motor end plate innervation in cWT and cKO mice on P7. *n* = 3–5 cWT, 5–6 cKO. Analyzed by two-way ANOVA followed by Sidak correction for multiple comparisons. **(E,F)** Representative images of tdTom + axons (red) innervating αBT labeled AChR clusters (green) in the thyroarytenoid (TA) muscle of cWT and cKO mice on P7. Insets show enlarged examples of neuromuscular junction morphology. **(G,H)** Representative images of tdTom + axons (red) innervating αBT labeled AChR clusters (green) in the posterior cricoarytenoid (PCA) muscle of cWT and cKO mice on P7. Insets show enlarged examples of neuromuscular junction morphology. **(I,J)** Representative images of tdTom + axons (red) innervating αBT labeled AChR clusters (green) in the lateral cricoarytenoid (LCA) and cricothyroid (CT) muscle of cWT and cKO mice on P7. All laryngeal muscle images shown are from tissue sectioned in the coronal plane (prepared along the length of the larynx in sections from dorsal to ventral). Scalebars = 50 μm in laryngeal muscle sections. The brightness and contrast of each channel was adjusted separately for visualization purposes.

Production of rodent USVs requires fine motor control of the muscles within the larynx (Riede, 2011, 2013). Laryngeal muscle contraction is driven by motor neurons located in the nAmb that project predominantly through the recurrent laryngeal branch of the vagus nerve (Wetzel et al., 1980; Nunez et al., 1985; Bieger and Hopkins, 1987; Daele and Cassell, 2009; Kelm-Nelson et al., 2018). To examine whether the loss of MET expression alters axon projections within the recurrent laryngeal nerve, neurofilament immunofluorescence staining was used to quantify the number of axons in recurrent laryngeal nerve cross sections collected on P7. Compared to control cWT mice, cKO mice exhibited an approximately 30% reduction in the number of axons traveling to the laryngeal musculature (Figures 3A–C), suggesting that vocal impairments resulting from *Met* deletion are the result of reduced motor input to the vocal organ.

To determine whether the loss of axons in the recurrent laryngeal branch results in the loss of motor endplate innervation to the intrinsic laryngeal muscles, cKO mice were bred to a conditional reporter line allowing visualization of tdTomato+ (tdTom+) axons. Laryngeal tissue sections were co-stained with fluorescently tagged alpha-bungarotoxin (αBT) to label acetylcholine receptor (AChR) clusters at the neuromuscular junction. The percentage of stained αBT profiles that were closely apposed to tdTom+ axons was then quantified in cWT and cKO mice on P7. In all of the intrinsic muscles in the larynx responsible for vocal production that were assayed (thyroarytenoid, TA; lateral cricoarytenoid, LCA; cricothyroid, CT; posterior cricoarytenoid, PCA; and alar portion of the thyroarytenoid, AlarTA), nearly all of the αBT labeled AChR clusters were juxtaposed by tdTom+ axons in cWT and cKO genotypes (Figure 3D), indicating that the remaining two-thirds of motor axons form laryngeal neuromuscular junctions following conditional deletion of *Met* (Figures 3E–J; AlarTA not shown).

Neuromuscular junction maintenance was also assessed in laryngeal samples from cKO mice at P60. Similar to the P7 time point, at P60 nearly all αBT labeled AChR clusters were closely apposed by tdTom+ axons in the intrinsic laryngeal muscles of both genotypes (data not shown), suggesting that the remaining axons continue to maintain neuromuscular junctions through early adulthood.

### MET Is Required for the Development of a Subset of Nucleus Ambiguus Motor Neurons

**FIGURE 4:**
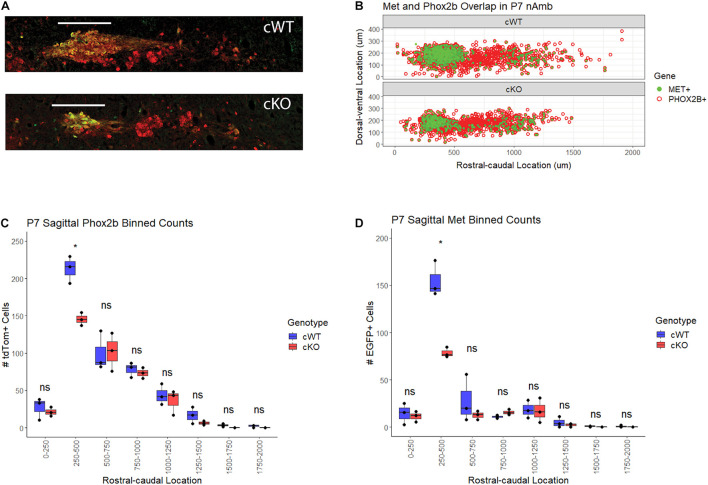
Topology of MET-EGFP expression and motor neuron loss in the nAmb following conditional deletion of *Met*. **(A)** Single-section representation of Phox2b + and MET + motor neuron topology in the nAmb. **(B)** Comparative topology of tdTom (Phox2b) and EGFP (MET) positive nAmb motor neurons. *n* = 3 per genotype. **(C)** Binned Abercrombie-corrected counts of TdTom + cells denoting Phox2b + motor neurons in nAmb of cWT and cKO mice. *n* = 3 per genotype. *t*-test, “*” indicates *p* ≤ 0.05. **(D)** Binned Abercrombie-corrected counts of GFP + cells denoting MET + motor neurons in nAmb of WT and cKO mice. *n* = 3 per genotype. *t*-test, *p* ≤ 0.05. Scale bars = 250 μm. The brightness and contrast of each channel was adjusted separately for visualization purposes.

To determine whether the loss of axons in the recurrent laryngeal branch of cKO mice is due to a reduction in the neurons that supply this branch located in the nAmb, the distribution of motor neurons along the rostral-caudal axis of the nAmb was examined. Previously published work from our laboratory demonstrated that MET expression is primarily confined to neurons located in the rostral compact and the caudal loose formations of the nAmb (Kamitakahara et al., 2017). To investigate structural changes in nAmb topology following conditional deletion of *Met*, cWT, and cKO mice were crossed with mice expressing a Cre-dependent tdTomato reporter and enhanced green fluorescent protein (EGFP) under the control of the MET promoter (*Met*^*EGFP*^), for which there is near absolute fidelity between EGFP and intrinsic *Met* expression (Kamitakahara et al., 2017; Kast et al., 2017). Because the *Met* promoter driven *Egfp* transgene (*Met*^*EGFP*^) is inserted independently from the *Met*^*fx*^ allele, this mouse model permits visualization and comparison of neurons expressing MET in cWT mice and neurons expressing non-functional MET protein in cKO mice (see section “Materials and Methods”). Using these mice, sagittal sections of the P7 brainstem were obtained and used to generate topologic maps of nAmb motor neurons. These maps confirmed previous findings from our laboratory showing that MET-expressing motor neurons are primarily confined to the compact and loose formations of the nAmb (Figures 4A,B). Very few MET-expressing cells were present in the middle portion of the nucleus, which corresponds to the semi-compact formation. This anatomical segregation of MET expressing motor neurons to the rostral and caudal ends of the nAmb also is present in cKO mice, demonstrating that the distribution of nAmb motor neurons is not altered in the absence of MET signaling (Figures 4A,B). In addition, qualitative comparison of the cWT and cKO maps highlight a prominent reduction in the size of the compact formation of the nAmb in cKO mice (Figure 4B).

To investigate the aforementioned qualitative changes in the nAmb after conditional deletion of MET, sagittal cell counts of all tdTom+ nAmb neurons from cKO were compared to those from the cWT mice. Quantification of tdTom+ neurons in cWT and cKO mice revealed a statistically significant 31.7% loss of motor neurons in the rostral compact portion of the nAmb (250–500 μm caudal to the facial nucleus) (Figure 4C). In agreement with tdTom+ cell counts, quantification of the EGFP+ subpopulation revealed a 49.2% loss of this motor neuron subgroup in the rostral nAmb (Figure 4D). The more caudal loose formation of the nucleus (500–1,500 μm caudal to the facial nucleus), where MET is also expressed, exhibited a trend toward a reduced number of neurons in cKO mice that did not reach statistical significance.

**FIGURE 5:**
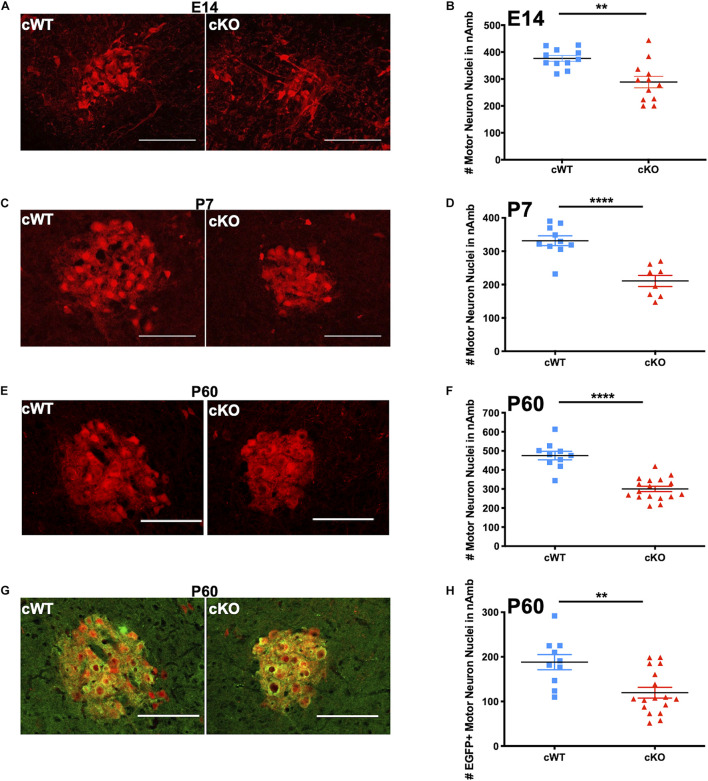
Analyses of lifespan loss of vagal motor neurons in the nAmb following conditional deletion of MET. **(A)** Representative images of the nAmb from cWT and cKO mice on E14.5. **(B)** tdTom + cell counts from cWT and cKO mice on E14.5. *n* = 11 cWT, 12 cKO. “**” indicates *p* < 0.01 as analyzed by parametric unpaired *t*-test. **(C)** Representative images of the nAmb of cWT and cKO mice on P7. **(D)** tdTom + cell counts from cWT and cKO mice on P7. *n* = 10 cWT, 8 cKO. “***” indicates *p* < 0.0001 as analyzed by parametric unpaired *t*-test. **(E)** Representative images of the nAmb of cWT and cKO mice on P60. **(F)** tdTom + cell counts from cWT and cKO mice on P60. *n* = 10 cWT, 17 cKO. “****” indicates *p* < 0.0001 as analyzed by parametric unpaired *t*-test. **(G)** Representative images of EGFP + (green) motor neurons (tdTom +; red) in nAmb of cWT and cKO mice on P60. Images in E and G demonstrate EGFP and tdTom overlap from the same samples. **(H)** EGFP + cell counts from cWT and cKO mice on P60. *n* = 10 cWT, 17 cKO. “**” indicates *p* < 0.01 as analyzed by parametric unpaired *t*-test. Scalebars = 100 μm. The brightness and contrast of each channel was adjusted separately for visualization purposes. Some images were cropped to center the nAmb in the image plane.

To verify the loss of MET-expressing neurons viewed in the sagittal plane of the medulla, we next processed additional sets of cWT and cKO mice to perform cell counts in nAmb imaged in the coronal plane. Analysis in this plane of section reduces potential variability in section orientations that can occur in the sagittal plane, particularly across multiple developmental timepoints. Brainstem coronal sections were collected at several developmental time points, beginning in embryonic life through adulthood in cWT and cKO mice. On E14.5, approximately 4 days after nAmb neurons are generated (Pierce, 1973), a decrease in the size of the developing nAmb in cKO samples already was evident (Figure 5A). Quantification of the number of neurons in the nAmb at this time point revealed a statistically significant 23.3% decrease in the number of tdTom+ neurons in cKO mice compared to cWT controls (Figure 5B). While the number of nAmb neurons was reduced, there were no ectopically located tdTom neurons evident in the embryonic and postnatal medulla, consistent with a lack of obvious deficits in cell migration. To determine whether this loss in neuron number is maintained later in life, tdTom+ cell counts were quantified in the brainstem of early postnatal and adult mice. Consistent with our earlier sagittal counts, there was a 36.4% loss of tdTom+ neurons on P7 (Figures 5C,D). The magnitude of neuronal loss in the nAmb remained consistent into adulthood, with a 36.9% decrease in tdTom+ neurons on P60 (Figures 5E,F). Together these data demonstrate that MET is required during embryonic and postnatal development for sustaining a normal number of nAmb neurons, particularly in the compact formation of the rostral nAmb.

cWT and cKO mice also were analyzed to quantify the neuron loss in the MET-expressing subpopulation of neurons in the rostral nAmb. To accomplish this, multi-transgenic mice were generated that express a Cre-dependent tdTomato reporter and the *Met*^*EGFP*^ allele to facilitate comparison of neurons expressing MET in cWT mice and neurons expressing non-functional MET protein in cKO mice (*Phox2b*^*Cre*^, *tdTomato*, *Met*^*fx*^, and *Met*^*EGFP*^ alleles; Figure 5G). Cell counts in the nAmb at P60 revealed a 36.4% decrease in the number of EGFP+ neurons (Figure 5H), consistent with the absence of a subpopulation neurons in the nAmb following deletion of *Met*. Furthermore, within each genotype, no sex differences were observed in the number of EGFP+ or tdTom+ neurons at any of the postnatal ages examined (Supplementary Figure 5-1).

### Overlap Between Phox2b^*cre*^ and MET-Expressing Cells Is Restricted to the Nucleus of the Solitary Tract, Dorsal Motor Nucleus of the Vagus, and Nucleus Ambiguus

To determine whether other brain regions were affected in our cKO model, whole brain samples from P2 pups and adult control mice expressing tdTomato in all Phox2b+ cells and *MET*^*EGFP*^ in all MET-expressing cells were systematically examined to investigate areas of *Phox2b*^*cre*^ and MET co-expression. Consistent with previous reports, vagal Cre recombinase expression in this *Phox2b*^*cre*^ line (designated line 3, JAX stock 016223) was observed in the nAmb, the dorsal motor nucleus of the vagus (DMV), the nucleus of the solitary tract (NTS), and the nodose-jugular ganglia (Scott et al., 2011). Phox2b-driven recombination was much broader than MET expression. MET was not expressed in the vagal sensory nodose-jugular complex. Within the nAmb, more than 34% of the neurons co-expressed Phox2b and MET. A much more limited subset of neurons in the DMV (∼6.7%) and NTS (∼3.0%) co-expressed Phox2b and MET. Examination of other brainstem structures revealed that there was minimal to no overlap between MET and Phox2b expression in the hypoglossal (XII), facial (VII), prepositus (PRP), and medial vestibular (MV) nuclei, or any other brainstem region (Supplementary Figure 6-1). In addition, no Phox2b+ neurons were located in the nucleus retroambiguus, consistent with other studies (Hernandez-Miranda et al., 2017). No overlap of MET and Phox2b was found in the cortex, hippocampus, hypothalamus, thalamus, nor periaqueductal gray. All forebrain regions examined exhibited minimal to no overlap between MET and Phox2b. The analyses demonstrate that in this conditional deletion model, *Phox2b*^*cre*^ and MET-expressing cells primarily overlap in nAmb, with minimal co-expression in the NTS, DMV. Thus, loss of *Met* in one or a combination of these nuclei are likely to be responsible for the severe vocal deficits.

**FIGURE 6:**
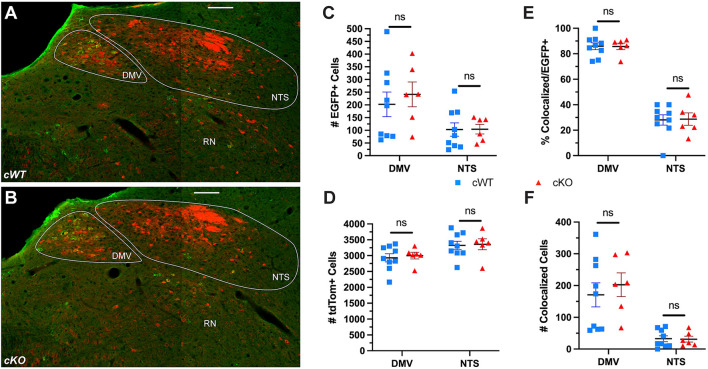
No difference in the number of neurons in the NTS or DMV is observed following conditional deletion of MET. **(A,B)** Representative images of the DMV, NTS, and reticular nucleus (RN) from cWT and cKO mice on P60. **(C)** EGFP + cell counts in the DMV and NTS from cWT and cKO mice on P60. *n* = 9 cWT, 6 cKO. No significant difference was found between genotypes using a non-parametric Mann-Whitney test. **(D)** tdTom + cell counts in the DMV and NTS from cWT and cKO mice on P60. *n* = 9 cWT, 6 cKO. No significant difference was found between genotypes using a non-parametric Mann-Whitney test. **(E)** Percentage of EGFP + cells co-expressing tdTom in the DMV and NTS from cWT and cKO mice on P60. No significant difference was found between genotypes using a non-parametric Mann-Whitney test. **(F)** Number of EGFP + cells co-expressing tdTom in the DMV and NTS from cWT and cKO mice on P60. No significant difference was found between genotypes using a non-parametric Mann-Whitney test. All scale bars = 100 μm. The brightness and contrast of each channel was adjusted separately for visualization purposes.

Our earlier analysis demonstrated that MET is necessary for proper nAmb development. To determine whether MET may be required for the development of neurons in the NTS or DMV, the number of EGFP+ and tdTom+ neurons was quantified. Colocalization of EGFP+ and tdTom+ neurons in the NTS are of particular interest, as a major cell loss in the NTS causes early postnatal lethality with impaired respiration and vocal production (Hernandez-Miranda et al., 2017). While the vast majority of EGFP+ neurons in the DMV also were tdTom+, only one third of EGFP+ neurons in the NTS were observed to colocalize with the tdTom reporter (Figures 6A,B,E). Cell counts further revealed that there are approximately 32 neurons, out of more than 3,300 tdTom+ neurons in the entire bilateral NTS, that express both MET and Phox2b (compared to ∼190 in the unilateral nAmb; Figure 6F). Additionally, the ∼3,300 tdTom+ neurons represent a small fraction of NTS neurons, as the vast majority of neurons in the NTS do not express tdTom in this model (Figures 6A,B). While it is very unlikely that this very limited number of Phox2b/MET expressing neurons in the NTS are responsible for the dramatic vocalization phenotype observed in cKO mice, we nonetheless quantitated cell numbers in cWT and cKO mice. There were no differences in the number of EGFP+ or tdTom+ cells in either the DMV or NTS between genotypes (Figures 6C,D). Additionally, there are no known connections between the DMV and the branchial organs involved in USV production in rodents. Together, these data analyses provide strong evidence that a selective and specific subset of vagal motor neurons located in the nAmb are lost due to *Met* deletion in cKO mice, indicating that developmental MET signaling is required by the neurons in nAmb that contribute to the circuitry underlying the production of USVs.

## Discussion

Like other motor circuits in the central nervous system, the present study demonstrates that the development of functional vocalization is dependent upon the timely exposure of neurons, and their responsiveness, to specific molecular cues (Purves, 1988; Jessell, 2000; Guthrie, 2007; Puelles et al., 2019). In the current study, we have newly identified MET as a critical neurodevelopmental signal that is necessary for establishing normal function and anatomy of the vagal motor nAmb. Specifically, the present data demonstrate that the loss of MET signaling results in enduring, major deficits in the ability to communicate using USVs, a significant decrease in the number of axonal projections within the recurrent laryngeal nerve supplying motor innervation of the larynx, and a significant decrease in a subset of vagal efferent motor neurons in the nAmb. The functional changes in the number of vocalizations are profound, with mutant mice exhibiting severe vocalization abnormalities. The functional analyses thus suggest that expression of MET in a subset of vagal motor neurons is necessary for proper USV production and the full repertoire of syllable shapes. Together with the anatomical studies across different timepoints, the experimental results reveal a relationship between USV production and developmental expression of MET by nAmb motor neurons.

### Deficits in Ultrasonic Vocalization in Early Development and Adulthood

The most dramatic phenotype observed in cKO mice is the severe disruption of ultrasonic vocal communication. This is a fully penetrant phenotype developmentally, as all cKO pups analyzed made few, if any, isolation-evoked USVs. Furthermore, analyses of repertoires demonstrated that when there is vocalization, the cKO pups produce only short, simple calls compared to the more complex long duration calls that are made by Cre- and cWT pups. Studies in which the recurrent laryngeal nerves are unilaterally or bilaterally transected demonstrate the importance of the connection between the nAmb and the larynx for vocal fold movement and USV production (Wetzel et al., 1980; Nunez et al., 1985; Bieger and Hopkins, 1987; Daele and Cassell, 2009; Kelm-Nelson et al., 2018). Additionally, the current study demonstrates that MET expression during development in a subset of vagal motor neurons of the nAmb is required for proper vocal function.

### Peripheral Innervation Abnormalities and Functional Deficits

Despite the loss of one third of the neurons in the nAmb and axonal projections within the recurrent laryngeal nerve in cKO mice, the remaining laryngeal motor end plates appeared to be innervated. Based on the functional analyses of vocalizations during development and in the adult, the remaining subset of nAmb-larynx connections are not able to mediate normal functions. This may be due to the primary loss of a subset of neurons in the nAmb, secondary maladaptive functional changes of presynaptic input onto the remaining nAmb neurons, and/or secondary inappropriate segregation of the functionally correct motor pools for vocalization. Innervation of the laryngeal musculature was examined in a small number of cWT and cKO mice expressing *MET*^*EGFP*^ on P7 (data not shown). This revealed that all of the intrinsic laryngeal muscles except the cricothyroid received innervation from *MET*^*EGFP*^ expressing axons in both genotypes, suggesting that if compensatory sprouting does occur it is supplied by the remaining EGFP+ neurons that survive following MET deletion. The approximate one fourth of adult cKO mice that exhibit normal vocalization indicate an adaptive process that can occur, but only sporadically. Thus, aberrant motor neuron targeting or non-functional sprouting could underlie what appears to be relatively normal structural innervation in the laryngeal muscle groups, and in some instances result in recovery of function. These are very interesting possibilities that will require extensive further anatomical, physiological, and functional experiments.

There are several relevant examples in humans that reflect aberrant laryngeal innervation. A neurological condition, synkinesis, involves co-contraction of different muscle groups that typically do not contract together. For example, during thyroid surgery, some patients experience recurrent laryngeal nerve injury. Subsequent non-selective reinnervation of laryngeal muscle groups can lead to synkinesis (Crumley, 1979, 2000; Flint et al., 1991). Synkinesis also can arise developmentally as demonstrated in mouse models of strabismus where chemokine receptor deletion has been shown to result in improper ocular muscle innervation by trigeminal motor neurons (Whitman et al., 2018). To our knowledge, no studies have specifically demonstrated laryngeal synkinesis of a developmental origin. However, HGF exhibits survival, growth promoting, and chemoattractive properties on the neuronal projections innervating the developing brachial arches (Ebens et al., 1996; Caton et al., 2000; Isabella et al., 2020). Therefore, the loss of MET expression in laryngeal motor neurons in the nAmb during development could allow for non-selective innervation by other motor neuron types in cKO mice and account for the observed vocal impairments. In addition, girls with Rett Syndrome may exhibit disrupted vagal motor circuit function, reflected in atypical feeding, swallowing, and vocalization (Morton et al., 2000; Einspieler and Marschik, 2019). This is of interest here, because *MET* transcription is attenuated by mutations in *MECP2*, and *MET* expression is nearly undetectable in postmortem brain samples of girls diagnosed with Rett Syndrome compared to matched controls (Plummer et al., 2013; Aldinger et al., 2020).

### Developmental Loss of a Subset of Nucleus Ambiguus Neurons

The MET receptor mediates a pleiotropic neurodevelopmental signal with well-established roles in neuronal proliferation and survival, chemoattraction, dendritic elaboration, synapse formation and maturation, and critical period plasticity (Nakamura et al., 1989; Ebens et al., 1996; Maina et al., 1998; Caton et al., 2000; Gutierrez et al., 2004; Lim and Walikonis, 2008; Judson et al., 2010; Lamballe et al., 2011; Avetisyan et al., 2015; Peng et al., 2016; Xie et al., 2016; Eagleson et al., 2017; Ma and Qiu, 2019; Chen et al., 2020; Isabella et al., 2020). Consistent with a developmental role, conditional deletion of *Met* in vagal motor neurons resulted in a statistically significant reduction in the number of neurons in the embryonic nAmb, detected just a few days after the onset of neurogenesis occurs in this population of cells (Pierce, 1973). Though we cannot completely exclude that some cells in the ventricular zone express MET, cell labeling is most evident in the mantle zone of the medulla, suggesting that MET expression is not involved in regulating the proliferation of these neurons (Supplementary Figure 1-2). This is consistent with our studies of the developing telencephalon (Judson et al., 2010) and those in the spinal cord that found the majority of MET expression to be restricted to non-proliferating cells (Ebens et al., 1996). We suggest that the prenatal phenotype is consistent with MET signaling being required for survival of a subset of nAmb motor neurons, the same type of survival heterogeneity exhibited by developing spinal motor neurons.

Virtually all motor neuronal populations are produced in excess during development, and then undergo a period of naturally occurring cell death (NOCD), eliminating up to half of these neurons between E11.5 and P1 (Hamburger and Levi-Montalcini, 1949; Hollyday and Hamburger, 1976; Hollyday et al., 1977; Purves, 1988; Oppenheim, 1991; White et al., 1998). The classic model of NOCD posits that competition for a limited supply of trophic factors produced by the target muscle serves as one of the central mechanisms through which apoptosis is regulated. Various trophic factors have been identified, including HGF, which protect specific neuronal subpopulations during NOCD. For example, there is a dose-dependent survival of lumbar motor neurons with increasing concentrations of supplemented HGF or chick muscle-derived extract (Ebens et al., 1996). While HGF supports the survival of lumbar neurons, it has little effect on brachial or thoracic motor neuron survival (Novak et al., 2000), demonstrating the specificity of trophic factor matching. We suggest that the differential MET-dependence for survival exhibited by different nAmb neuron subpopulations is consistent with the findings from spinal motor neurons, lending additional support for the data reported here. Previous studies from our laboratory demonstrate expression of HGF at nAmb target innervation sites in the muscles of the esophagus and larynx at E13.5 and E15.5 (Kamitakahara et al., 2017), positioning it as a likely candidate mediating survival or other developmental functions in this subpopulation of nAmb neurons.

While loss of MET signaling results in a reduction of neurons in the compact formation of the nAmb, we demonstrate that the survival of other subsets of EGFP+ neurons are not impacted. The mechanism for subset-specific neuronal survival in the nAmb of cKO mice is not known, but the current results are consistent with subset-specific loss of neuronal populations observed in other developmental trophic factor interactions. For example, GDNF-deficient mice exhibit a loss of approximately one quarter of spinal lumbar neurons despite expression of the GDNF receptors, Ret and Gfra1, in all spinal motor neurons (Moore et al., 1996; Oppenheim et al., 2000). Similarly, in addition to expression of MET, the nAmb has been reported to express receptors for a number of other trophic factors, including p75NTR, TrkA (Koh et al., 1989; Gibbs and Pfaff, 1994) TrkB (Liu and Wong-Riley, 2013), TrkC (Helke et al., 1998), Lifr (Li et al., 1995), Cntfr (MacLennan et al., 1996), gp130 (Nakashima et al., 1999), and Gfra1 (Mikaels et al., 2000). Here it is possible that the remaining EGFP + neurons that express non-functional MET in cKO mice are able to maintain their survival through expression or upregulation of receptors for other trophic factors. Yet the surviving nAmb neurons have limited ability to replace functionally the loss of the subset of MET-expression neurons. Further studies focusing on combinatorial trophic factor expression and their impact on laryngeal connectivity and neuronal survival will inform a greater understanding of vocal motor circuit formation and mechanisms through which developmentally or surgically induced deficits in vocal function arise.

## Data Availability Statement

The original contributions presented in the study are included in the article/Supplementary Material, further inquiries can be directed to the corresponding author/s.

## Ethics Statement

The animal study was reviewed and approved by the Institutional Animal Care and Use Committee of The Saban Research Institute, Children’s Hospital Los Angeles.

## Author Contributions

AK, RA, H-HW, and PL: conceptualization. AK and RA: methodology. AK, RA, AL, and VM: formal analysis, investigation, and visualization. AK: writing—original draft. AK, RA, AL, VM, H-HW, and PL: writing—review and editing. AK and PL: supervision and project administration. AK, PL, and AL: funding acquisition. All authors contributed to the article and approved the submitted version.

## Conflict of Interest

The authors declare that the research was conducted in the absence of any commercial or financial relationships that could be construed as a potential conflict of interest.

## Publisher’s Note

All claims expressed in this article are solely those of the authors and do not necessarily represent those of their affiliated organizations, or those of the publisher, the editors and the reviewers. Any product that may be evaluated in this article, or claim that may be made by its manufacturer, is not guaranteed or endorsed by the publisher.
