# Supplementary material for: MET Receptor Tyrosine Kinase Regulates Lifespan Ultrasonic Vocalization and Vagal Motor Neuron Development
Source: Front Neurosci. 2021 Nov 4;15:768577. doi: 10.3389/fnins.2021.768577 (PMC8600253; doi:10.3389/fnins.2021.768577)
Supplement: Supplementary file 1 [file Data_Sheet_1.docx]

Supplementary Material


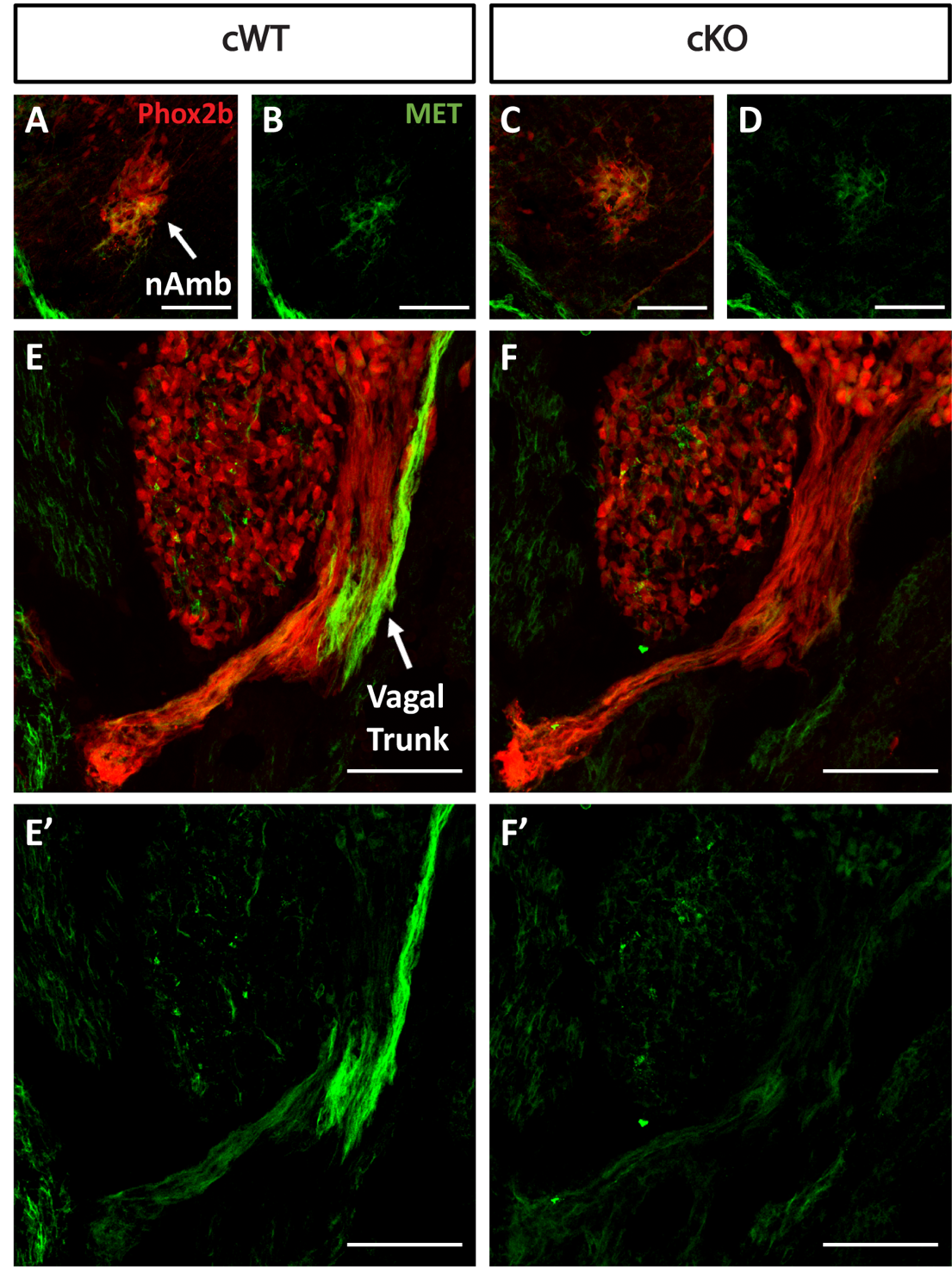


**Supplementary Figure 1-1. Conditional deletion of *Met* results in early embryonic loss of MET protein in vagal motor neuron axons.**

In the cKO mouse model, Cre-mediated recombination facilitates excision of exon 16 of the *Met* allele (the functional ATP binding site), such that only truncated, non-functional protein is transcribed (Huh et al., 2004). To confirm loss of MET from vagal motor neurons, immunohistochemistry was used to examine protein expression in vagal axonal projections. While no antibodies are available to exclusively detect the portion of the receptor transcribed by exon 16, studies from our laboratory and others have demonstrated the signaling incompetence and rapid degradation of MET protein following Cre-mediated recombination in *Met^fx/fx^* mice (Huh et al., 2004; Judson et al., 2010; Peng et al., 2016). In agreement with these results, in cWT mice, abundant MET immunolabeling (green) could be visualized in both the nAmb and axonal projections of developing Phox2b+ vagal motor neurons (tdTomato, red) on E14.5 (A, B, E, and E’). By contrast, in cKO mice, MET immunolabeling could only be detected in the nAmb cell soma, but not in axonal projections, consistent with truncated MET protein being degraded in the cell soma and not transported to innervation targets (C, D, F, and F’).

All scalebars = 100μm. n = 4 mice per group.

The brightness and contrast of each channel was adjusted separately for visualization purposes.


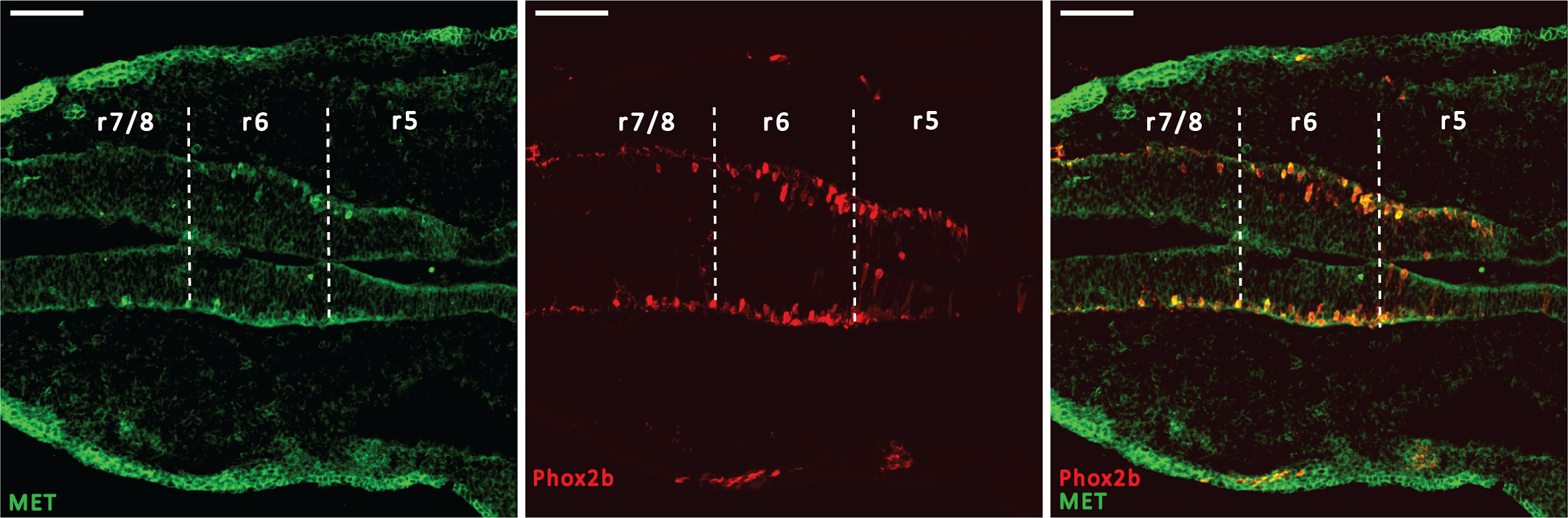


**Supplementary Figure 1-2. Early Cre-mediated recombination driven by the Phox2b promoter in the developing brainstem.**

The Cre-dependent reporter tdTomato (tdTom) was used to identify all vagal populations following functional deletion of MET. On embryonic day (E) 9.5, tdTom+ neurons were present in rhombomeres 7/8, the region from which vagal neurons originate (Lumsden and Keynes, 1989), indicating that recombination and deletion of MET occurs early, just following cell birth (Pierce, 1973). Representative images show MET protein immunoreactivity (green) and tdTom endogenous fluorescence (red) on E9.5. r5, rhombomere 5; r6, rhombomere 6; r7/8, rhombomeres 7 and 8. Scale bars = 100μm. The brightness and contrast of each channel was adjusted separately for visualization purposes.


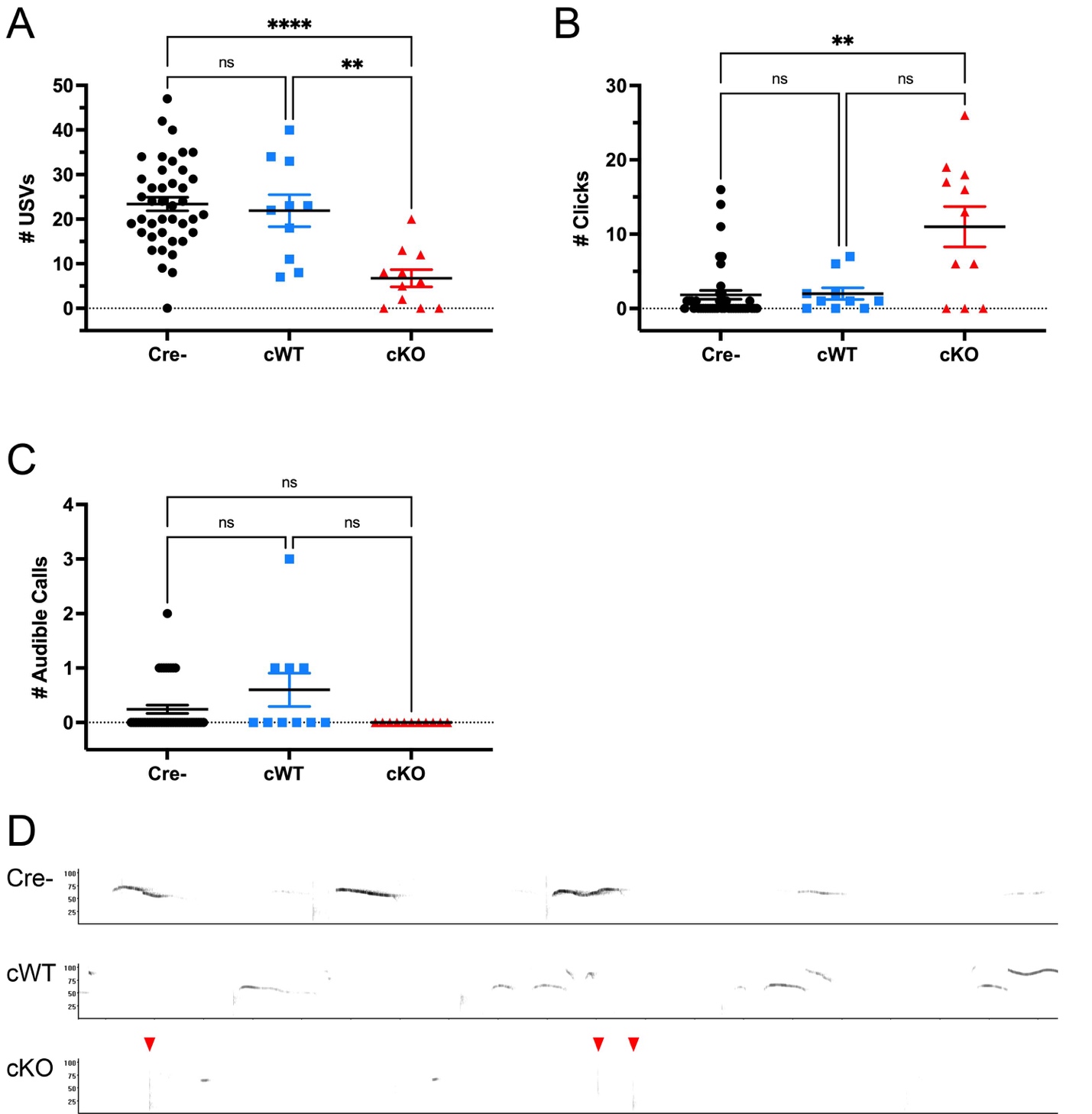


**Supplementary Figure 1-3. Mechanical stimulation assay to directly elicit calls.**

Audio recordings were made on P7 following a tail pinch stimulus. The number of USVs (A), clicks (B), and audible calls (C) were quantified for each genotype. Despite high motivation to vocalize (pain), cKO pups made significantly fewer calls in the ultrasonic range compared to Cre- and cWT littermates, and no calls in the audible range. However, cKO mice did produce significantly more clicks than Cre- pups, suggesting that emitted clicks may be generated by cKO pups in place of USVs. (D) Illustration of representative, one second spectrograms from P7 Cre-, cWT, and cKO mice following the tail pinch. Red arrowheads indicate clicks emitted by cKO pups.


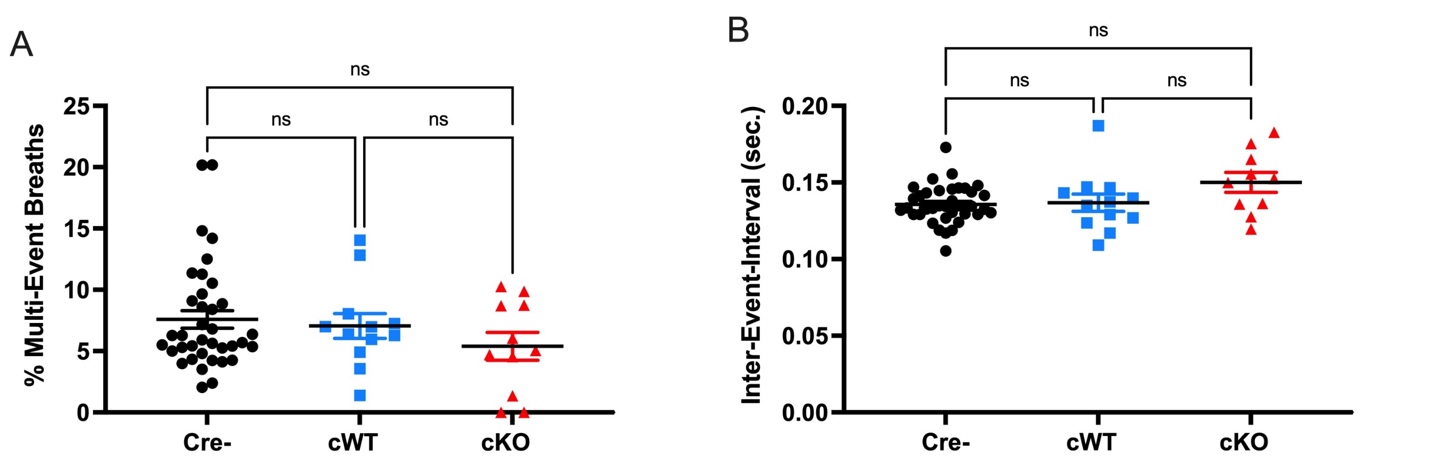


**Supplementary Figure 1-4. Respiratory pattern during vocalization.**

Audio recordings made on P7 to examine isolation evoked USVs were subsequently analyzed to examine respiratory pattern during bouts of vocalization. The percent of breaths containing more than one call or click (e.g. event) was not significantly different between genotypes (A). Furthermore, the time between each call was not affected by conditional MET deletion (B).


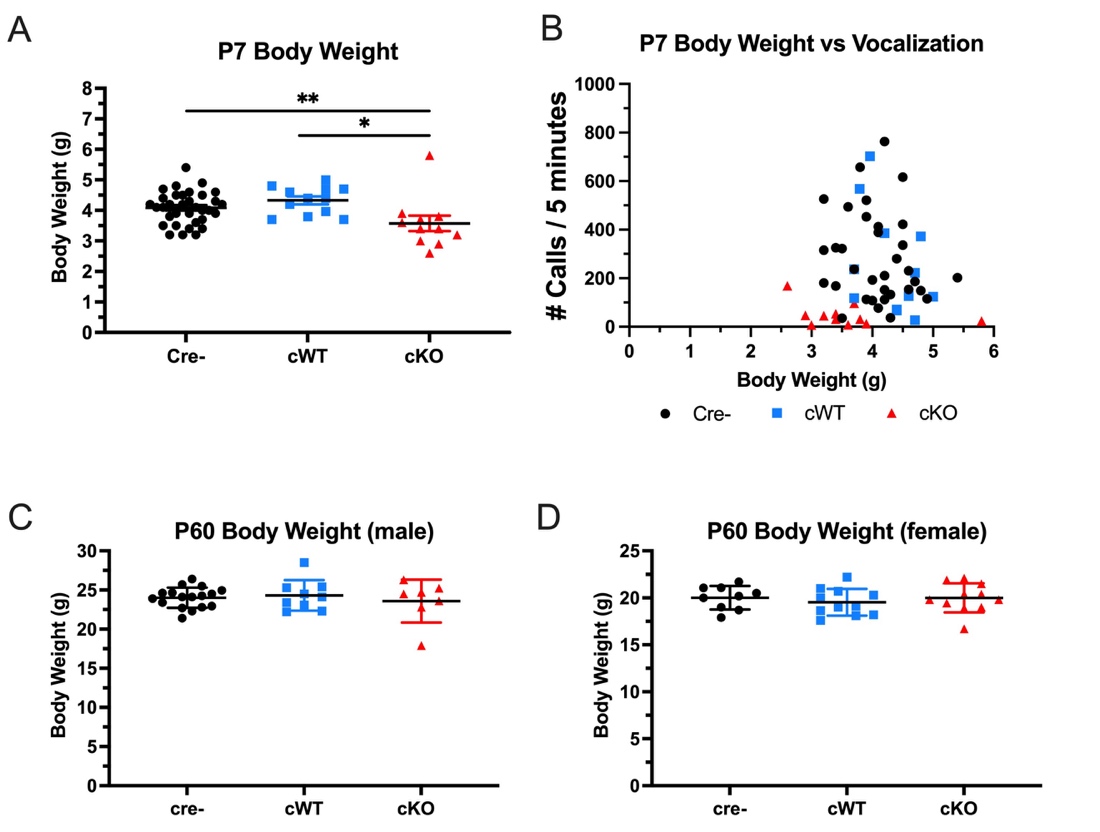


**Supplementary Figure 1-5. Body weight does not correlate with vocalization disturbances observed following MET deletion.**

(A) Quantification of body weight in grams (g) of mouse pups from all genotypes on P7. n = 34 Cre-, 12 cWT, 11 cKO. Analyzed by one-way ANOVA with Tukey correction for multiple comparisons.

(B) Scatterplot of body weights vs the number of calls recorded during the 5-minute test period. n = 34 Cre-, 12 cWT, 11 cKO

(C) Quantification of body weight in grams (g) of male mice from all genotypes on P60. n = 17 Cre-, 9 cWT, 7 cKO. Analyzed by one-way ANOVA with Tukey correction for multiple comparisons.

(D) Quantification of body weight in grams (g) of female mice from all genotypes on P60. n = 9 Cre-, 11 cWT, 11 cKO. Analyzed by one-way ANOVA with Tukey correction for multiple comparisons.


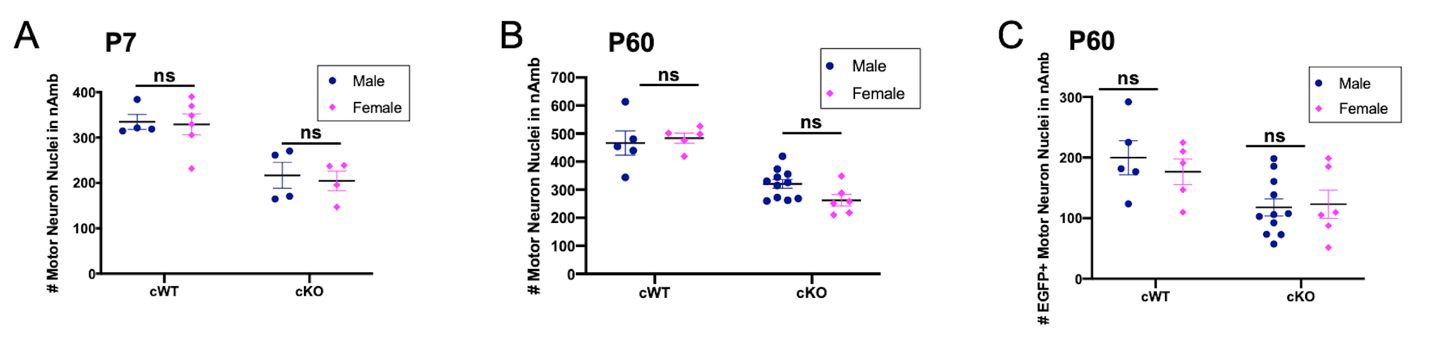


**Supplementary Figure 5-1. Reduction of nAmb neurons in cKO mice is not different between sexes.**

(A) tdTom+ cell counts from cWT and cKO mice on P7, by sex. n = 4 cWT males, 6 cWT females; n = 4 cKO males, 4 cKO females. ‘ns’ indicates no significant sex effect as analyzed by 2way ANOVA.

(B) tdTom+ cell counts from cWT and cKO mice on P60, by sex. n = 5 cWT males, 5 cWT females; n = 11 cKO males, 6 cKO females. ‘ns’ indicates no significant sex effect as analyzed by 2way ANOVA.

(C) EGFP+ cell counts from cWT and cKO mice on P60, by sex. n = 5 cWT males, 5 cWT; n = 11 cKO males, 6 cKO females. ‘ns’ indicates no significant sex effect as analyzed by 2way ANOVA.


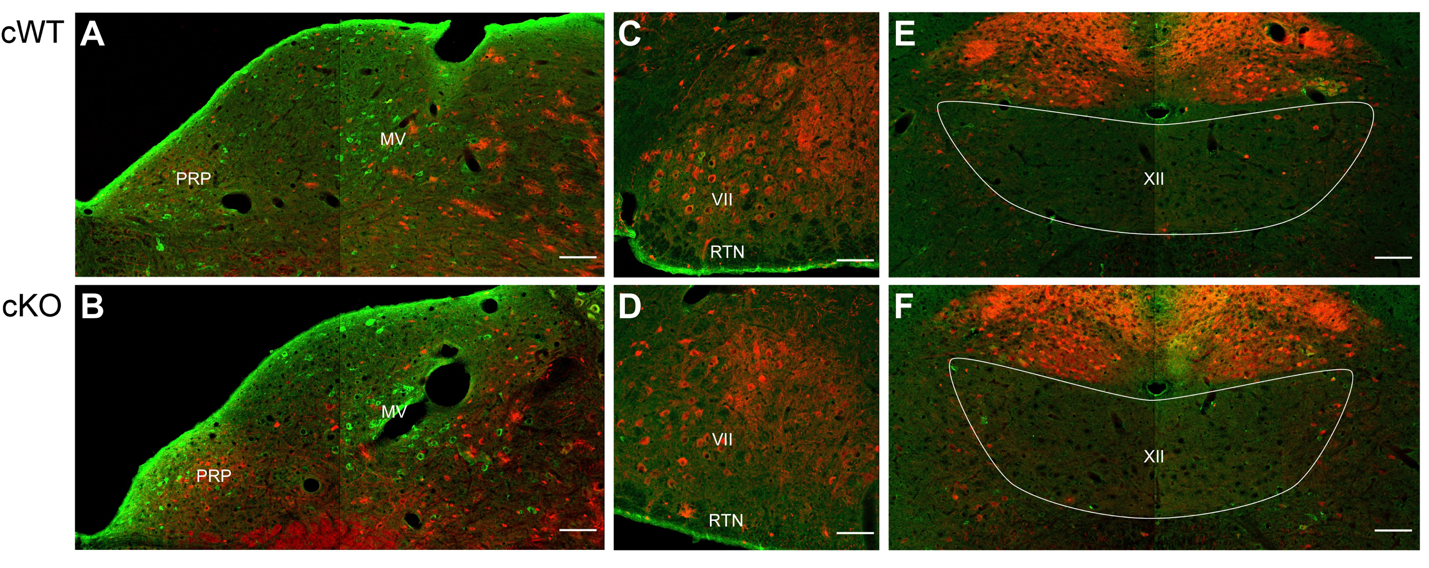


**Supplementary Figure 6-1. Limited colocalization of MET and Phox2b outside of the vagal nuclei.**

(A, B) Representative images of the nucleus prepositus (PRP) and medial vestibular nucleus (MV) from cWT and cKO mice on P60.

(C, D) Representative images of the facial (VII) and retrotrapezoid (RTN) nuclei from cWT and cKO mice on P60.

(E, F) Representative images of the hypoglossal nucleus (XII) from cWT and cKO mice on P60.

All scalebars = 100μm. The brightness and contrast of each channel was adjusted separately for visualization purposes.


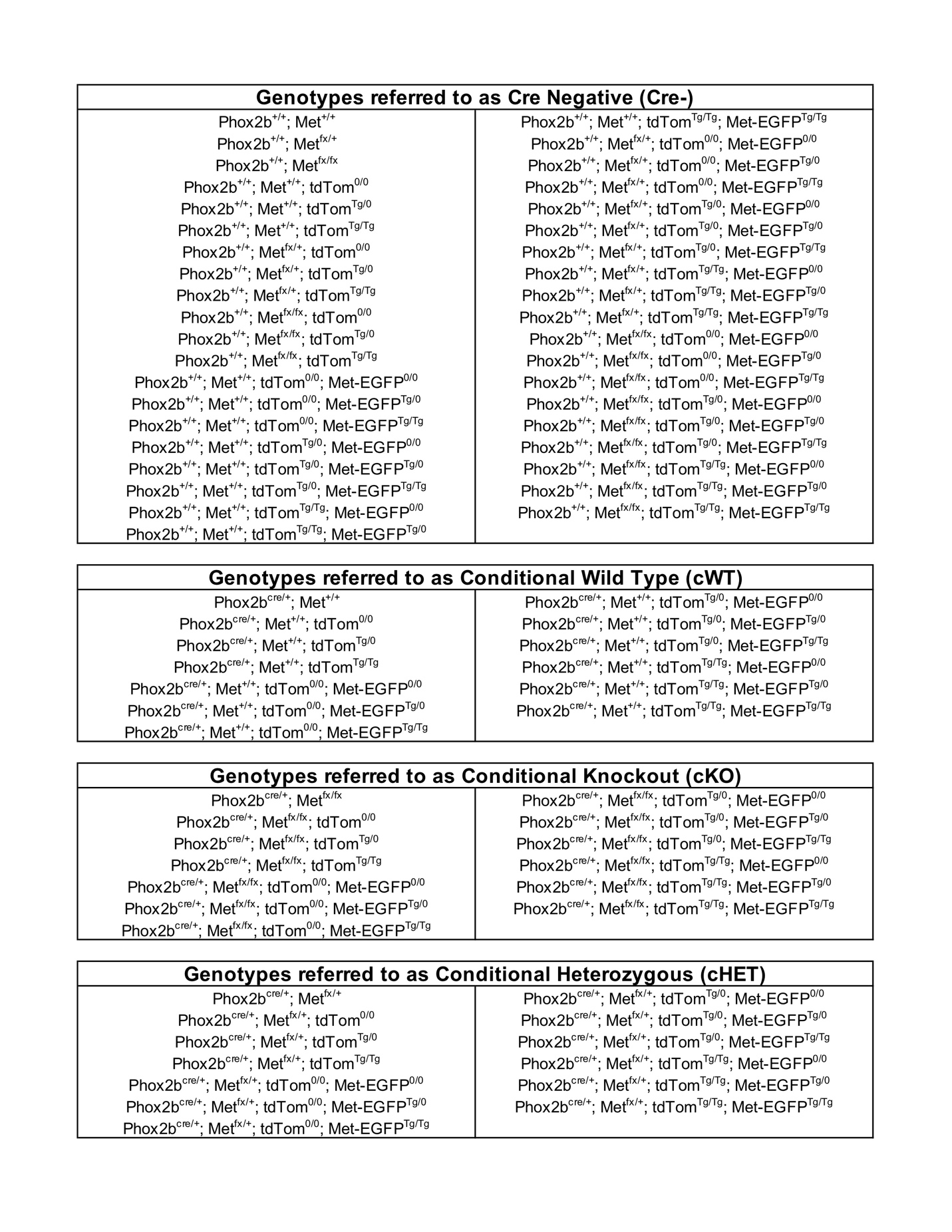


**Supplementary Table 1. Simplified Genotype Categories.** To simplify the genotype names of mice used, Cre- is used to describe mice lacking the *Phox2b^cre^* allele, conditional wild type (cWT) is used to describe any mouse with the *Phox2b^cre^* allele but lacking the *Met^fx^* allele, and conditional knockout (cKO) is used to describe mice with the *Phox2b^cre^* allele and two copies of the *Met^fx^* allele. ‘+’ indicates the presence of the wild-type allele. ‘cre’ indicates the presence of the Cre recombinase allele. ‘fx’ indicates the presence of the floxed *Met* allele. ‘0’ indicates the absence of a reporter allele. ‘Tg’ indicates the presence of a reporter allele.
